# Psychology of Spinoza
*Benedicti de Spinoza Opera quæ supersunt omnia. Ed. C. H. Bruder.


**Published:** 1857-04-01

**Authors:** 


### Art. VI.—PSYCHOLOGY OF SPINOZA.*

BY PROFESSOR HOPPUS, LL.D.

Benedict de Spinoza, whose place in the development of Cartesianism is usually assigned next to Malebranche, was born of Jewish parents, at Amsterdam, in 1632. He devoted himself, at an early period of his life, in recluse solitude, to the reading of the Old Testament, and the Talmud; and he deeply studied the Cabbala, or esoteric philosophy and criticism of the Rabbis; but he soon shocked his friends by the novelty and boldness of his speculations. He was summoned before the heads of the synagogue, and on their failing to convince him that he was in error, he was ordered to leave the assembly. He now cultivated the society of some Christians, and professed to prefer their creed to Judaism. Van den Ende, a physician, who taught him Greek and Latin, had a daughter who was an excellent musician, and appears also to have been a learned young lady, for she greatly assisted Spinoza in his studies. Unfortunately, she taught him, at the same time, Latin and something more; for she unconsciously inspired an ardent passion to which she did not respond; and she soon yielded to the more attractive suit of a wealthy young merchant from Hamburgh, carried on with pearl necklaces, rings, and other ornaments. Spinoza, like a true philosopher, consoled himself by study; and, henceforth, according

* Benedicti de Spinoza Opera quæ supersunt omnia. Ed. C. H. Bruder.

to his biographer, devoted himself wholly to the cultivation of his mind, and remained unmarried to his death.* The Latin which he had learned was useful to him, as he wrote his works in this language, in a condensed and energetic, though by no means an elegant style.

After relinquishing theology, having met with the works of Descartes, he read them with avidity, adopted the general method of their author, and ever after acknowledged his obligation to him for all he knew of philosophy. He now ceased all connexion with the Jews, his former co-religionists, and never more set foot in the synagogue. It has, indeed, been asserted, that he formally adopted the Christian faith; but this statement appears to have no foundation in truth. He held many conversations with learned Mennonites, and with other intelligent men of various Christian communions, and he changed his name from *Baruch* to its Latin synonyme Benedict; but he never joined any other religious profession, after his secession from his paternal faith, though he sometimes attended the Lutheran worship, and highly valued good sermons. His renunciation of Judaism so alarmed the Rabbis, who feared the effect of his example, that they offered him a pension of a thousand florins if he would only consent to attend the synagogue, as before—being willing apparently to connive at his heterodoxy, if he would but pay this hollow compliment to their faith. Spinoza received the proposal with scorn, indignantly declaring that he would not frequent the synagogue again, "if the pension were tenfold" the proffered amount.

He had now produced a feeling which nearly proved fatal to his life. One evening, as he was passing by the old Portuguese synagogue, in Amsterdam, he received the thrust of a poniard, which, fortunately, did him no mischief; but, finding himself no longer safe in the city, he retired to some distance in the country. He was now publicly charged with abandoning Judaism, and was solemnly anathematized with the most awful ceremonies. Before the assembled synagogue, a vast number of candles made of black wax were lighted, the tabernacle containing the books of the law was opened, the officiating minister on an elevated seat uttered, in a doleful tone, the solemn curse; while the sound of the trumpet† gave effect to the extraordinary scene. The candles were then gradually extinguished in a large

* *Vide* Boullanvilliers.

† Salomon Maimon was threatened with the Shofar (trumpet) at Altona; and on his saying that it was a mere buckshorn, the Rabbi fell to the ground with horror. "Der Rabbi zeigte ihm den Schofar, mit den finstern Worten; 'weisst du was das ist?' " Als aber der Schüler Kants sehr gelassen antwortete, 'es ist das Horn eines Bockes,' da fiel der Rabbi rücklings zu Boden vor Entsetzen." *Der Salon*, von H. Heine. 2ter Band. s. 115.

tub filled with blood; and, finally, the whole assembly, inspired with horror and execration, re-echoed the deep "Amen."

Notwithstanding the tendency of his system, Spinoza appears to have been a man of blameless morals, both at the time of his excommunication, and during his subsequent career. In the failure of all his prospects in life, he betook himself to the art of polishing glasses for telescopes, microscopes, and other optical instruments. His fame, as a clever optician, even reached the ears of Leibnitz, who wrote to him in consequence. In this way Spinoza gained a very humble subsistence; but, as his wants were few, he found it enough. In his twenty-eighth year, he retired to Rheinsburg near Leyden, where he pursued his trade as an optician, and continued his studies.

The extraordinary sensation which the writings of Descartes had made in the reading world caused frequent discussions and disputes; and, at the earnest request of his friends, Spinoza resolved to write on the new philosophy. In 1663, he published, at Amsterdam, *Renati Descartes Principia Philosophiæ*, with an Appendix on his *Meditationes;* in which work he abridged the speculations of Descartes in an accurate and intelligible manner, so that no better digest of the original Cartesianism is anywhere to be met with. We have here, also, the germs of Spinoza's posthumous work, entitled *Ethica;* and, indeed, the principal points of his peculiar system. The impression made by this book was anything but favourable to the author's reputation among his opponents, much as it raised him in the estimation of his friends; and he now retreated to Voorburgh, near the Hague. All admitted his great ability, but many of the admirers of Descartes were annoyed at some of his criticisms on their master's opinions. He afterwards went to live at the Hague; and here he was in no little danger from the popular displeasure, as he was foolishly suspected of being a spy, at the time of the invasion of Holland by the French. About this period of his life many traits of disinterestedness and generosity occur. He refused to be made heir to a considerable property, because he thought it unjust to the relatives of the testator; he gave up to his sisters his own portion of his paternal estate; and he declined the offer of a pension made to him by the Prince de Condé, on condition that he would dedicate one of his works to Louis XIV., saying that he was "indebted to no one but to God." Some of the anecdotes of his Spartan abstemiousness and superiority to animal indulgence are curious. According to his account-books, his dinner one day cost him about two-pence farthing; but, on another day, having indulged himself with a few raisins extra, it amounted to the sum of two-pence halfpenny!

Spinoza's second work, and the only other published in his lifetime, came out at Hamburgh, in 1670, entitled *Tractatus Theologico-Politicus.* This is a treatise on the relation between religion and politics. In this book he boldly endeavoured to emancipate human thought from the yoke of authority, and strenuously advocated the doctrine which has since been called the "right of private judgment." He maintained freedom of religious opinions, though by a sort of Hobbism, he invested the sovereign with powers incompatible with such liberty; and he says that no religion is obligatory, excepting so far as it has the sanction of the Government, for "it is by princes that God reigns on the earth." He maintains that the State ought, compulsorily, to regulate the form and observances of the Church, though under an outward uniformity all sorts of creeds should be admitted. He deprecated all political changes, and holds that every nation ought to maintain the form of government which it has been used to. Some of the advice he elsewhere gives to rulers in reference to the consolidation of their power, is more worthy of the satrap of an Oriental Monarch than of a philosopher, and seems quite at variance with his own mild and gentle character.* In his bold theological speculations, he anticipated much of the rationalism which has prevailed in Germany since the latter part of the last century. Not only has his general system proved the seed of the varying pantheisms which have marked the speculative philosophy of the Germans, since the sceptre passed from the hands of Kant; but his rationalism, as has been remarked by one of his recent biographers, has even "anticipated the Hegelian Christology, which, in the hands of Strauss, Feuerbach, and Bruno Bauer, has made so much noise in the theological world."†

In 1673, the Elector Palatine offered to Spinoza the chair of philosophy at Heidelberg; but he declined it, on the ground that he should not be able to avoid causing religious controversy by his lectures. It took many years before the Christian faith was so undermined in Germany, that the University of Heidelberg was prepared for the teaching of Paulus. For more than twenty years Spinoza had shown a tendency to consumption; of which disease he died, at the Hague, in 1677, in his 45th year. The amiableness of his character, and the care he sometimes took to encourage religious observances and feelings in others, appeared

* Tractatus Politicus, cap. xviii.

† "Biographical History of Philosophy," by G. H. Lewes. Vol. III. p. 125. In a letter to Oldenburg, Spinoza says:—"Dico ad salutem non esse omnino necesse Christum secundum carnem noscere; sed de æterno illo filio Dei, hoc est, Dei æternâ sapientiâ, quæ sese in omnibus rebus, et maximè in mente humanâ, et omnium maximè in Christo Jesu manifestavit, longe aliter sentiendum." Opera Posthuma, Spin. p. 450. Ibid.

remarkable, considering the usually admitted tendency of his philosophical system. He taught children the external duties of religion; questioned his host and hostess respecting the edification they had derived from the sermons they heard; and, on the last day of his life, which was Sunday, he would not allow them to be absent from church on account of his illness. Even those who most revolted from his speculations, allowed that he was distinguished for his temperance, quietness, and blameless conduct. He sometimes remained in doors for months together, diligently employed in writing, and in making lenses. His chief recreation is said to have been smoking his pipe, and watching the contests of spiders with flies, and among themselves—an amusement which, oddly enough, so tickled his fancy, that it often made him laugh till the tears rolled down his cheeks.

The different estimates which have been formed of the opinions of Spinoza, appear to have arisen not only from the previous system, philosophical and theological, of the readers of his works, but in a considerable measure from the obscurity which not unfrequently attaches to his language. Compare many parts of his "Treatise on the Improvement of the Understanding,"* with Descartes' "Meditations," or his "Discourse on Method," and we soon find a difference in practical clearness, though we recognise the like simplicity of aim in seeking for truth. Voltaire says:—"You are very confused, Benedict Spinoza, but are you as dangerous as they say? I think not; and my reason is that you are very perplexed; you have written in bad Latin, and there are not ten persons in all Europe who will read you from end to end. When is an author dangerous? When he is read by the idle of the court, and by women."† It would be difficult to find a more candid or enlightened critic than Jouffroy, one more competent or more disposed to do justice. He says:—"that there is nothing contradictory in the system itself, is not my opinion. I am obliged to confess that, after the most attentive study I have been able to give it, there are several portions of the system which still leave me in doubt."‡ Every one who has seriously sat down to read Spinoza's works must have felt inclined to sympathize with this statement of the talented and laborious philosopher, who, during his short life, was one of the greatest ornaments of the French Eclectic School, by no means inferior, as seems to us, to the founder himself. Another reason of the different estimates which have been made of Spinoza's opinions,

* De Emendatione Intellectus.

† Encyclopédie Française. Art. "Dieu."

‡ Lectures on Ethics: a translation from the French, by G. Ripley.—This is one of the most detailed and luminous accounts of Spinoza's opinions that we have met with. The only thing which deteriorates from its value is, that it does not contain quotations.

may arise from the fact of some of his readers having derived their knowledge of them from the works published in his lifetime, in which they were not fully developed, and others from his great work, the *Ethica*, which, as we have remarked, was posthumous. The opposition which he had to encounter induced him, sometimes, to endeavour in some measure to accommodate himself to existing opinions, as appears in his *Tractatus Theologico-Politicus*, and in his correspondence. It is to the *Ethica* that we must look for the detailed exposition of his matured views. This is his most important posthumous work. The others, all of which are unfinished, are *De Intellectus Emendatione; Tractatus Politicus;* and a *Hebrew Grammar*. His *Letters and Answers*, also, were published after his death. In the work entitled *Ethica more geometrico demonstrata*, we find Spinoza's system fully developed in a learned and elaborate attempt to prove the existence of the Deity as the sole being in the universe. This treatise is written in a strictly geometrical form, and its author has, on this account, been called the "Euclid of metaphysicians;" but the brief, rigid, and mathematical way in which he has chained his doctrines together, is not exactly adapted to the greatest clearness on a subject so far removed from geometry. His fragment above-named, on the Improvement of the Intellectual Faculty, though not published till after his death, was an early production of his pen. It was intended as an introduction to the discovery of truth; and it is evident, from his notes to the work, which are numerous, that the author contemplated a lengthened treatise, though we have only a tract of thirty-six pages, with his editor's remark, *Reliqua desiderantur*. This work was designed to lay the foundation of a true method of philosophy, in a criticism of the facts of consciousness. He first inquires into the nature of *good*. He concludes, from observation, that the things which men in general regard as constituting the *summum bonum* may be reduced to three—wealth, honour, and pleasure—all of which distract the mind from the true good. That is true good which leads towards perfection. The *summum bonum* is to reach this perfection; and it consists in the cognition of the union which the mind has with all nature. Here we see the germ of Spinoza's future pantheistic development. In order to lead society in general to this perfection, he relies on moral philosophy, education, medicine, mechanic art, and above all on some remedy for the mind (*modus medendi intellectum,*) in order that it may be purified from error, and attain to truth. This end is to be promoted by philosophers always speaking popularly (*ad captum vulgi*), by encouraging a sanitary temperance, and a very moderate pursuit of wealth and all bodily good.

There are, according to our author, four modes of knowing (*modi percipiendi*): by hearing or perceiving any sign, which includes testimony; by general experience (*experientia vaga*): *e. g.*, "that oil nourishes flame;" by inference (*ubi essentia rei ex aliâ re concluditur*), as when, from our sensations, we infer that the soul is united to the body; and by essential cognition (*ubi res percipitur per solam sui essentiam*): as, for instance, "by actual knowledge, I know what knowing is;" or when I perceive that "two and three are five," or "that parallels to a third line are parallel." The best method of knowing is attained by a strict acquaintance with our own nature and faculties; by carefully noting the differences, agreements, and oppositions of things, and what are their properties, and what not; and by ascertaining the limit of man's power to reach perfect knowledge. True ideas are the mind's instruments. The intellect by its native energy forms to itself these ideas. Indeed, like some schools of the later Germans, Spinoza supposed the mind capable of grasping the essential nature of things by its ideas, coming face to face with *being* itself, by means of an intellectual intuition—an exaggeration this, of Descartes' appeal to consciousness and its "clear ideas." Leibnitz was far from being wrong in calling Spinoza's philosophy *un Cartesianisme outré*; it is so even in some of its elementary positions.* Our author next treats of fictious, false, and doubtful ideas, which may float in the imagination or the memory, in distinction from ideas which are true and adequate, as being the ideas of the reason. In pursuing his method, he next discusses the conditions of definition. If the thing to be defined is a created thing, the definition must comprehend the proximate cause—*e. g.*, a circle is defined, "a figure which is described with any line one extremity of which is fixed, the other moveable"—the circle is the result of the conditions. Again, a conception or a definition of a thing requires that all the properties of the thing may be concluded from what is stated of it. Thus the equality of the radii, and all other properties, are determined by the construction, which, as above, defines the circle. The definition of an uncreated thing involves the conditions—that it requires no object beyond itself for its explication; that its definition includes its existence; that it is not explicable by any abstract ideas; that all its properties should follow from the definition. Spinoza adds that a definition, in order to be called perfect, must explain the intimate

* Spinoza said, "Certainty is nothing but the objective essence itself of the object. The true idea of Peter is the objective essence of Peter. So that, in order to arrive at certain knowledge, we want nothing but the true idea. We reach the highest certainty when we have the adequate idea or objective essence of anything; that is to say, certainty and the objective essence are the same thing." *De Emend. Intell.* vi. We have here an *Identitäts-lehre* by anticipation.

essence of the thing, and must avoid employing properties as a substitute for this: thus, he says, we are not unfolding the essence of the circle when we say the radii are all equal, but only naming a property. The author closes his fragment by treating of the powers and properties of the intellect. It certainly knows things *as they are.* It can form ideas absolutely; and it can deduce them from others. The ideas which it forms absolutely express infinity, which is necessary to the idea of the finite. Positive ideas are formed before negative ones. Clear and distinct ideas so arise from the laws of our nature, that they *seem* to depend on our own power. From this brief sketch of the contents of the work *De Intellectus Emendatione*, the reader may in some measure judge of Spinoza's method; and if he reads this brief treatise for himself, he will perhaps find that it is not always very lucid. We certainly miss very often, in the pages of Spinoza, the perspicuity and clearness of his master Descartes, with whom we are far enough from always agreeing; but we certainly less frequently find him obscure.

In the *Tractatus Politicus*, which Spinoza wrote shortly before he died, we meet with clear trains of thought, and a luminous style. He treats of polity in general; and of monarchy, aristocracy, and democracy, as forms of government. In respect to the limit and functions of the ruling power, as regards the relation of academical education to the state, he reminds us of Adam Smith. He objects to endowments, as calculated to repress rather than to foster genius; and he holds that science can only flourish by its teachers relying entirely on their own resources, and earned emoluments. We are disposed to think that if Spinoza had lived in England, in the nineteenth century, he would have been obliged to acknowledge that his theory on this subject required a little revision, however disposed he was to content himself with Spartan fare and "single blessedness."

Though his method of seeking truth was so much that of Descartes, he soon regarded the method as capable of a more extended development, landing in very different results. In one of his letters,* in answer to the inquiry—what is the best mode of advancing with certainty in the road to the knowledge of things? Spinoza replies (agreeably to the main principles of his Treatise on the Intellect),† that "the best method is that in which we can direct and chain together our clear and distinct perceptions (ideas); for the intellect is not, like the body, liable to vary with circumstances (*casibus obnoxius*), on this account alone—namely, that one of these clear and distinct perceptions, or several together, are adapted to be absolutely the cause of another clear and distinct perception; nay, all our clear and distinct percep-

* Epist. xlii. † De Intellectus Emendatione.

tions cannot arise otherwise than from other clear and distinct perceptions which are in us, and which admit no other cause out of ourselves.* Hence it evidently appears what ought to be the true method, and in what it chiefly consists—namely, in the simple cognition of pure intellect (*in solâ puri intellectus cognitione*), and in its nature and laws; and in order that this method may be acquired, it is necessary above all things to distinguish between intellect and imagination, or between true ideas and others—that is, those which are fictious, false, and doubtful, and absolutely all which depend on memory alone.†

It appears from these statements that Spinoza agreed with Descartes as to the importance to be attached to "clear and distinct ideas and perceptions," as the basis of real knowledge; though these ideas and perceptions led him away from the dualism of his predecessor (who believed in two sorts of substances, matter and mind) to the dogma that there is no real being in the universe but the τὸ ἓν καὶ τὸ πᾶν. This theory of ideas and their validity has been more or less elaborated by Descartes and Leibnitz, as well as Spinoza, and we may add Malebranche, but especially by the three former; for Malebranche's main stand-point, the theosophic vision, tended to shorten the process of testing the psychological subjectivity of our ideas. With Descartes, Spinoza made it a principle to admit nothing as true, the grounds of which he did suppose himself distinctly to recognise. But he carried his theory farther; he said, not only is everything true that we clearly and distinctly cogitate, but every true idea is also *adequate*, that is, it agrees perfectly with its object, and the more perfect the object, the more perfect is the idea. This axiom, that the true idea, or real knowledge, must wholly agree with its object—must seize, as it were, upon the very essence of that object, lies at the foundation of all Spinoza's axioms and first principles. They are to be seen and known to be true by an *intellectual intuition*. Like Descartes, therefore, Spinoza appealed to consciousness giving a clear and distinct testimony, as the final criterion of truth: our clear and distinct ideas and perceptions, as being freed from all uncertainty, are expressions of what truly and really is. But it is instructive to reflect to what different results this theory of *ideas*, so similarly treated by Descartes, Spinoza, and Leibnitz, conducted these several renowned philosophers. Descartes and Leibnitz, it must be admitted, signally departed from their own rules as soon as they advanced beyond the threshold of their speculations, and gave to the world some most gratuitous theories; and if Spinoza was more consequent, more consistent throughout with the principles he first laid down, he was, never-

* Quod quidem ex hoc solo constat, etc. Epistol xlii. † Ibid.

theless, led to conclusions which seem to offer, in some respects, still greater violence to reason! Surely these "clear and distinct ideas" require to be scrutinized with some jealousy, and guarded with more practical care from the intrusions of imagination, than we find exemplified in much of the philosophy of either of these celebrated men.

Considering that Spinoza was a Cartesian as to his adoption of the mathematical or deductive method, which he followed much more rigorously and formally than Descartes himself, and that he was altogether of the *à priori* school of philosophers, it is the more to be noted that he should find any distinct point of coincidence between his own notions of psychological speculation and those of Lord Bacon, whom he criticises in his Correspondence as not only having erred widely, along with Descartes, "from the true knowledge of the first cause and origin of all things," but also as "not having known the true nature of the human mind," and as "never having reached the true cause of errors," because they failed of a right estimation of "*ideas*" as leading to knowledge. Yet, with Bacon, he described the line of speculation he proposed to follow as being independent of any theory of the nature of mind itself. The Baconian principle, as exemplified by Bacon and the metaphysicians of his school, was to seek for properties and phenomena only, since natures and essences are beyond our ken; and Spinoza himself says that, for the purpose of distinguishing the pure cognitions of the intellect from the ideas of mere imagination and memory, "we need not know what the mind is in its cause," but only digest the history of its perceptions according to Lord Bacon's method; (*more illo quo Verulamius docet.*)

The main problem which Spinoza proposed to himself may be stated as follows:—Given the notion of substance, as it is conceived by reason, and expressed by a proper definition—to derive from this notion all that is involved in it, by a rigorous deduction, apart from experience; and then, having obtained such logical deduction, to put it in the place of the universe of being. With this aim, Spinoza adopted as much as possible the procedure of the geometrician, endeavouring to apply it to his metaphysics of matter and of mind. The fundamental idea of the geometer is the abstract notion of space; the fundamental idea of the philosopher, according to Spinoza, is to be that of *substance.* As clear and distinct ideas, supposed to be attained, lie at the basis of his theory of knowledge—so the idea of substance, which is said to be one of them, is the corner-stone of his metaphysical, cosmological, and theological system. The reader will judge for himself, as we advance, to what extent the whole fabric of Spinozism is built upon ambiguities and assumptions, sincerely as Spinoza himself believed that all was irrefragable.

He gave the name *Ethica* to the work which developes his main doctrine, because he regarded the whole of true philosophy as closely identified with human virtue; and virtue, as being in its principle the love of God, must have its origin in the true knowledge of him.* The first book of the *Ethica* is entitled *De Deo sive Infinito.* The second *De Naturâ et Origine Mentis, seu de Finito*, deduces from the previous idea of God that which we should form respecting man as a thinking being. The third is *De Naturâ et Origine Affectuum*, or the sources and mechanism of the passions, a topic which he regards as involving all the phenomena of man. The fourth is *De Servitute Humanâ, seu de Affectuum Viribus;* and here the author endeavours to show that there is a necessary order of development in human nature, and that the will also is under necessity. The fifth and last book is entitled *De Potentiâ Intellectus, seu de Libertate Humanâ*, in which he proposes to show the nature and operation of free-will.

The work sets out with eight definitions:—I. Cause of itself (*causa sui*) is that, the essence of which involves existence; or that, the nature of which cannot be conceived of but as existing. The idea here evidently is that of necessary and self-existence. II. The *Finite* is that which can be limited or bounded by another thing of the same nature. In this way body and thought may be limited, though not by each other. III. *Substance* is that which is self-contained, and is conceived of *per se;* or that, the conception of which does not require to be formed from the conception of anything else. IV. *Attribute* is that which the understanding perceives as constituting the very essence of substance. V. *Modes* are the affections of substance, by means of which we can conceive of it. VI. *God* is a Being absolutely infinite, the substance which consists of infinite attributes, each of which indicates an infinite and eternal essence. VII. That is said to be *free*, which exists solely by the necessity of its nature, and which is determined to action by itself alone. That is *necessary*, or constrained, which owes its existence to something else, and which acts from certain and determinate causes. VIII. *Eternity* is existence itself, so far as it is conceived necessarily to follow from the mere definition of an eternal thing.—It is evident that the scholastic way in which these definitions are expressed, is in itself sufficient to throw over them a certain air of obscurity. Their meaning, however, is perhaps for the most part sufficiently intelligible; and if the wording of some of them may be questioned—after all, definitions do not prove anything, and an author may be allowed to abide by his own.

Seven axioms follow. I. Everything which exists, exists either in itself or in something else. II. Whatever cannot be conceived

* Amor Dei non nisi ex cognitione ejus oritur. Tractat. Theol. Pol. IV. 42.

of through another thing (*per aliud*) must be conceived of *per se.* III. From the cause, the effect necessarily follows; and there can be no effect without its cause. IV. Knowledge of the effect depends on knowledge of the cause, and involves it. V. Things having nothing mutually or in common, cannot be understood through each other; that is, the conception of the one does not involve the conception of the other. VI. A true idea must agree with its prototype, or representative in nature (*cum suo ideato*). VII. Whatever can be conceived of as non-existent, does not involve existence in its essence.—These axioms may, with little difficulty, be admitted. The first means that everything must be either self-existent or not. The fourth has been much condemned: among others, by Mr. Hallam, who thinks that this axiom is the seat of a fundamental fallacy, and remarks that "the relation between a cause and effect is surely something different from our perfect comprehension of it, or, indeed, from our having any knowledge of it at all; much less can the contrary assertion be deemed axiomatic."* Mr. Lewes, on the contrary, defends the axiom as true according to the Spinozistic sense of it. It is not meant that no effects are manifested to us, of which we do not also know the causes; but that a complete knowledge of the effect can only be had through a complete knowledge of the cause. "If you would know the effect in its totality, you must also know the cause in its totality. The antecedent was once only a sequent to its cause. To penetrate the mystery, you must know *what* the effect is, and *how* it is; you must know its point of departure and its point of destination."† We have little doubt that the above remarks furnish the proper clue to Spinoza's meaning.—In order to illustrate the manner in which he applies his definitions and axioms to prove his doctrines, we will introduce a few examples from his propositions:

I. Substance is prior in nature to its affections or accidents: Def. 3 and 5.

II. Two substances with different attributes have nothing in common with each other: Def. 3.

III. Of things having nothing in common, one cannot be the cause of the other:‡ Axiom 5; Axiom 4.

IV. Two or more different things are distinguished from each other, either by the diversity of their attributes or by that of their modes (*affectionum*): Axiom 1; Def. 3 and 5; Def. 4.

* Lit. of Europe, vol. iv. p. 246.

† Biog. Hist. of Philosophy, vol. iii. p. 135.

‡ This proposition is the old Greek fallacy, "like cannot act on or produce unlike;" which daily experience contradicts, for our sensations do not resemble outward objects which produce them; but the principle favoured Spinoza's theory, that there is only one substance in the universe.

V. In the nature of things, there cannot be two or more substances of the same nature or of the same attribute: Prop. IV., Def. 3 and 6.

VI. One substance cannot be produced by another substance; (Prop. V. II. III.; Corollary 1). It follows that substance cannot be produced by anything else, (Axiom 1; Def. 3 and 5; Corollary 2). The contradictory of this proposition is absurd; Axiom 4; Def. 3.

VII. It belongs to the very nature of substance to exist: Coroll. to Prop. VI., and Def. 1.

VIII. All substance is necessarily infinite: Def. 2; Prop. V. Scholium 1. It follows alone, from proposition VII., that all substance must be infinite: Scholium 2.—Here Spinoza anticipates objections to Prop. VII.; but there would be no difficulty, he says, if men did not confound the divine with the human—if they would only understand by substance that which exists in itself—that, the knowledge of which does not require the knowledge of anything antecedent; in short, if they would only attend to the Definitions and Axioms, they would then be convinced that the existence of substance is an eternal truth, and that only one substance of the same nature can exist.

We have now given a sufficient specimen of our author's method in the *Ethica*. In a similar way he treats all his propositions, adding their demonstrations, which we have only indicated, for want of space.* The whole work is digested in the most rigid order, each new theorem depending on the definitions, axioms, and preceding propositions, and each demonstration meriting, as the author believed, the triumphant attestation of the three letters, Q. E. D. Whoever sits down seriously to study these pages of Spinoza, will indeed find that they require even a more wakeful and painstaking attention than a book of geometry; for in these abstract metaphysics we can have no aid from diagrams—to say nothing of the entire conviction which attends every link of a chain of geometrical demonstration.

We will now proceed to some further development of Spinoza's system, keeping as nearly as may be to his own expressions. God is the one infinite substance, holding potentially, in his own self-existent being, the whole universe. He is *causa sui*—that is, "his essence involves his existence;" for a cause of or in itself is that, the nature of which cannot but be conceived of as existing.† This *causa sui* is, therefore, the *sole substance*, or that existence which is in itself, and is conceived of by means of itself, since the conception or notion of it does not require any other conception from which it must be framed. It is the abso-

* For the same reason we have not quoted long extracts from the original Latin.

† Eth. Part. I. Def. I.

lute, the independent, the highest and only reality. Of course there can be but one such substance, and it is necessary and infinite ;* nor can we imagine another to produce it. We must not understand our author, therefore, to use the term "substance" in the ordinary sense. According to him, matter or body is only one mode in which the Deity is manifested. Both matter and spirits are nothing but manifestations of Him, the one and only real substance ; for all else is finite, and dependent on His being.

Attribute is "that which our intellect perceives to constitute the essence of substance ;" and modes are the affections of substance, by which the attributes are manifested in something else, through which manifestation the mode is conceived of.† The formal definition given of the Deity is: "the Being absolutely infinite, or the Substance consisting of (*constantem ex*) infinite attributes, every one of which expresses an eternal and infinite essence." This substance exists necessarily, and is "absolute affirmation," one and indivisible. Extended existence and thinking existence are not two different substances, but only attributes, or modifications of the attributes of the one divine substance,‡ in which all things exist, apart from which nothing can be conceived of, and to the necessary activity of which they all owe their origin.§ And as the Deity is constrained by the laws of His own nature alone, He is the *immanent* but not the *transient* cause of all things ; that is, all finite things are but the necessary emanation of the divine nature, and not productions created in time, and dependent on single acts.|| Nothing, therefore, is contingent ; all is eternally determined by the necessary laws of the divine substance. Hence God is *natura naturans*,—an expression used by Bacon in reference to the supposed forms or essences of matter, but which Spinoza uses to signify the Deity in respect to His being regarded as a free cause (*quatenus ut causa libera consideratur*). The term *natura naturata* is applied to all things as existing in the Divine Being, since they flow from the very necessity of his nature, and are but modes of his attri-

* Ibid. Def. V. Prop. VI., VIII. This is very like some statements of Descartes, and might well be developed from them. He says (Principia, Part. I. 51), "that which truly exists is substance, or that which requires no other thing to its existence. There can be but one such being, namely, God ; all other things only exist by his co-operation. Hence the name *substance* does not univocally belong to him and to them."

† Ibid. Def. IV.

‡ Vel attributa vel affectiones attributorum. *Vide* Prop. VIII. Schol. 1. Prop. XIII. Schol. Prop. XIV. Corol 1. Descartes said that the essence of matter is extension ; Spinoza ascribes to matter infinity, necessity, unity, and indivisibility. Prop. XV. Schol. He also says, consistently with this, that if any one part of matter were annihilated, all extension would vanish with it. Epist. IV. ad Henr. Oldenburgh.

§ Prop. XV., XVI. || Prop. XVIII., XXV.

butes.* For while God is spoken of as a "free cause," this only means that we are wont to regard him as such: the system denies the Divine freedom, in any other sense than that of unlimited natural power, or the absence of obstruction or constraint: God does not act from liberty of will: things could not have been made by him otherwise than they are. Our author goes on to identify as one the divine intellect, will, and power, saying that they are "essentially one and the same thing." As to intentional motive principles, or final causes, these are denied. The Deity does not act on the principle of *what is good* (*sub ratione boni*); for, if so, "there would be something good beyond Himself, and not dependent on him—something for Him to copy from or aim at." He acts without any such impulse, and simply by the mere necessity of His own energy and power. This very power is His "essence," by which Himself and all things are and act; for by the necessity of His essence He is cause of Himself and of all things (*causa sui et omnium rerum*).†

Spinoza next treats of the ideas which the human mind should form respecting itself as well as of all finite things. All is to be identified with the one and only substance, as being the same with it (*imo una et eadem substantia est*), sometimes comprehended under one, sometimes under another attribute. Thus the "*mode*" of extension, and an idea of that mode, are but one thing expressed in two ways: a circle existing in nature, and the idea of it, are one and the same thing developed by means of different attributes.‡ Things and the ideas of things are equally the necessary result of the same order and connexion. Things come of necessity from the Divine existence; ideas come from the Divine thought. God is the cause of every idea of a finite mind,—not merely as origin and container of all things, but also in so far as His infinite understanding is considered as modified by the idea of a particular finite being (*quatenus alia rei singularis idea affectus*).§ "Hence it follows that the essence of man is constituted by certain modifications of the attributes of God; for there is something in that essence which is in God, and which cannot even be conceived of without him."—"The essence of man, therefore, is an affection or mode which, in a determinate manner, expresses the nature of God."||—"It follows that the human mind is a part of the Divine intellect (*partem esse divini*

* Prop. XXIX. Schol.

† *Vide* Ibid. Prop. XXXII. Corol. 1. Prop. XXXIII., XVII. Schol. Prop. XXXIV.

‡ This, in the original, might seem Malebranchianism, did we not know how much more it meant Circulus in natura, et idea circuli, quæ etiam in Deo est. Eth. Part. II. Prop. VII. Schol.

§ Prop. VIII. and IX.—Ibid.

|| Ibid. Prop. X Cor.

*intellectus*); and when we say that the human mind *perceives* this or that, we say, in short, that God has this or that idea—not in respect that He is infinite, but so far as He is developed through the nature of the human mind, and constitutes its essence."* Again: agreeably to the general theory, (according to which infinite extension and infinite thought belong to the one universal Being), the body and the mind of man, being only modes or finite determinations of the infinite development of God, are one and the same thing, conceived of sometimes under the mode of extension, sometimes under that of thought.† This doctrine of identity is evidently cosmothetic idealism—and not like materialistic pantheism, ancient or modern.

As to those ideas of the mind which are the final vouchers for truth, Spinoza compares them with the ideas of God. Now, "all the ideas which are in God are true, for they all agree, altogether, and adequately with their objects: so every idea in ourselves, which is absolute or adequate, is true." But when the mind is only determined, accidentally, to contemplate a thing, independently of the inward bond which that object has to the universal Being, a confused idea is the result. Every error arises from inadequate ideas of the imagination; but the *reason* always gives adequate ideas, and is always true. We always know when we have a true idea, and we cannot doubt of it; for truth is the criterion both of itself and of falsehood. Our cognition of the eternal and infinite essence of the Deity, which every true idea involves, is adequate and perfect. Hence the infinite essence and the eternity of God cannot but be known to all.‡ Truly, if human reason possesses such an intuition, we ought long ago to have mastered all the sciences!

The directly moral part of Spinoza's system is contained in the three last books of the *Ethica*. His moral philosophy, in which man is viewed in those absolute relations which reason discerns, is pure and elevated, symbolizing with that of the Stoics. Frederic Schlegel, who certainly had no tendency to Spinozism, even estimates his ethics as having a considerable advantage over those of the Porch. Nevertheless, his moral *theory* is sometimes expressed in language which evidently identifies it with the views of the sentimental and utilitarian schools. He regards moral good and evil as "nothing more than our modes of thinking, or the notions that we form;"§ and he expressly makes utility the same thing with goodness.|| His doctrine of human volition amounts to downright fatalism; and the same may be said in

* Ibid. Prop. XI. Cor. † Ibid. Prop. XXI. Schol.

‡ *Vide* Ibid. Prop. XXXII., XXXIV., XXIX., XXXIV., XXXV., XLV., XLVI., XLVII.

§ Eth. Part. IV.

|| Per bonum id intelligam quod certo scimus nobis esse utile.—Ibid. Def. I.

regard to his views respecting the Divine will. He holds that "no mind has absolute or free will;" and that the mind of man is determined to will this or that, by a cause which is again determined by another, and this again by another, and so on *in infinitum.* For as mind is a certain and determinate mode of thinking, it cannot be an absolutely free cause of its own actions. There is, in the mind, no absolute power of understanding, desiring, willing, or the like."* Indeed, will and intellect, says our author, are one and the same thing—"they are identical with singular volitions and ideas—a singular volition and a singular idea are one and the same." And "as thought and will are one, so the knowledge of good and evil are nothing but the affection of joy and sorrow, when we are conscious to ourselves of the one or the other."†

Every being, says our author, desires, by the necessity of its nature, to continue in the condition for which its nature adapts it. God necessarily exists; he therefore necessarily desires to remain in existence; and as this existence is universal and unlimited by any other, he is absolutely happy as he is absolutely perfect. Man, as a development of the Deity, partakes of the desire of God to exist, and to remain in existence. Of the soul of man, knowledge is the main element; and the desire of continued existence is therefore identified with the desire to extend and increase knowledge. Spinoza characterizes human desire as being this appetency of continued existence as an intelligent being. This desire, however, meets with obstacles, as well as with objects for its gratification. Hence the relation of man's condition (which is variable) to this elementary desire, produces pain or joy, hatred or love, hope or fear. These Spinoza calls *passions*: they are "secondary feelings;" the desire of continued intelligent existence, alone, is primary and fundamental. Our author's reason given for this distinction is, that the passions or secondary emotions of the mind arise from the agency of external causes, which act on us in a wholly passive state; while the general and abiding desire of continued intelligent existence is original, innate, and independent of external causes.‡

The above quotations and references will enable the reader to judge of the chief characteristics of Spinozism. Its main drift is to prove that there is, in the universe, but one substance, which is God, endowed with infinite attributes—those best known to us being infinite extension and infinite cogitation. But these two are really one: extension is visible thought, and thought is visible extension; they are the objective and the subjective, of

* Eth. Part II. Prop. VIII.
† Ibid. Prop. XLIX. Corol. et Scholium finale.
‡ Eth. Part IV.

which God is the identity. All things exist but as modifications of this Divine Substance; apart from him they are nothing. He is their cause, by the eternal necessity of his nature—their cause by dwelling in them, not by creating them as distinct existences from himself; so that, in all events, physical and moral, he is at once cause and effect, agent and patient. Everything termed matter is a mode or manifestation of the Divine attribute of extension; every thought, desire, emotion, volition of man, is a mode or manifestation of the Divine attribute of thought. Descartes, in some places of his works, makes extension and thought identical with material and spiritual substance, respectively: Spinoza says they are attributes of the one only substance, for they exist only *per aliud*, not *per se*. Thought does not think, extension itself is not extended; thought and extension are attributes of the one thinking, extended substance.

There are apparently *three* kinds of existence:—First, attributes, properties, or qualities, and effects or phenomena; but all admit that we can only conceive of these in relation to substances, independently of which they have no existence. Secondly, other things seem more real: man, who has certain properties of body and mind, appears to be a substance; but, as neither minds nor bodies originated without a cause foreign to them, and cannot continue their own existence, their being is only that of accidents—it is not truly real: their independent existence is only apparent. Thirdly, though the above two kinds of existences are all that experience makes known to us, reason can go further—can go beyond derived, accidental, and dependent existence, and can perceive that there must be somewhere a Being whose existence is the essence of the former, and who is self-existent. We now reach the only real Being (*Ens realissimum*), the one only substance; for reason will not allow us to suppose more than one self-existent, invisible Being, containing all existence in himself—in ancient phrase, τὸ ὂν—from whose existence all his infinite attributes necessarily flow.

As there is but one real substance, and as we cannot conceive of substance but as extended, and there is infinite extension in the universe, extension is an infinite attribute of the Infinite Being. Spinoza does not seem to have brought forward any demonstration of the necessity of the attribute of infinite thought, as he has of that of extension, which he considers inseparable from substance. He holds, however, that thought is another infinite attribute of the one substance, agreeably to the ancient doctrine of Parmenides and his school, that thought is only an aspect of τὸ πᾶν or the One-all. Indeed, we find, in the ancient Greek metaphysics, some striking resemblances to the doctrines of our author ; especially in the opinions of Xenophanes ;* though Spinoza arrived at his conclusions more exclusively by the "high *à priori* road," than the celebrated chief of the Eleatic school. It does not appear, however, that Spinoza was influenced by any study of the purely Greek philosophy ; but much of his system has a strong resemblance to some of the Cabbalistic dogmas, which were of Pantheistic tendency.

As God is the only substance, it follows that all things must exist in him and through him, as their inherent cause. The whole universe is only a manifestation of his being. If we loosely talk of other beings or substances, all that is or ought to be meant by this language is, that they are *modes* in which his attributes are manifested. According to our author's doctrine, there is no divine volition in these manifestations. As the ancient Greeks sometimes talked of Jupiter himself being subjected to inexorable fate ; so Spinoza's God is irrevocably bound by an iron necessity, as a perfection of his nature. He is little else than an infinite, omnipotent machine, acting by laws which are not controlled by anything that can in strictness be termed wisdom ; for wisdom implies intelligent choice and freedom ; but in this system no room is left for such a moral power. Spinoza ridicules the notion commonly entertained, that the Deity wills and acts for a certain end, as though it were possible for him to prefer some other end, or any end at all. All his actions are rigidly determined by the laws of his own nature ; and free-will, in the moral sense, is not one of them. All we know of God is his thought, and his extension ; we cannot attribute *will* to him, as separate from thought, and as capable of a varying determination. Not that it is denied, in terms, that God is free—nay, he is the only free agent ; but his free agency consists only in this, that he is free from being influenced to act by any other nature or power than his own. Man's acts depend on his circumstances, on the will of others, and ultimately like all other agencies they must be traced back to God : man, therefore, is not a free agent, because dependent. God is free from dependency on any other being ; but he wills not from design, and has no passion or disposition analogous to man's. His will is a mere spontaneity of his nature ; all is unchanging, unalterable, eternal necessity. So far as intelligent freedom is concerned, an automaton chess-player would represent Spinoza's God.

It is remarkable that while he strenuously maintains that all finite existences and their acts are the natural and inevitable expression of the necessary laws of the divine nature—only so many phenomena of deity, he, nevertheless, energetically repels

* Who was described as "lost in the One-all," (*εἰς ἓν ταὐτό τε πᾶν ἀνελύετο*). *Vide* Sextus Empiricus ; *Institut. Pyrrhon.* I.

the allegation that he confounds the universe with God. He denies that it is God; it is a mode in which his attributes are necessarily manifested. God is the eternal living principle of all things; the material world is one phase of his infinite attribute of extension. Now, the modes of manifestation are finite; but the one Substance, or God, is, in all respects, infinite. His attributes, too, are, in themselves, infinite, each in its own way: it is only as being many that they are finite, one limiting another; and each expressing, under one mode only, the Divine essence or existence. But surely the finiteness of the "modes," and the reciprocal limitation of the "attributes" (which latter, in some places, he identifies with modes) do not, in Spinoza's theory, separate them from the "one and only Substance." There is in the system as close an identification of the universe with God as there is, in the current psychology of man, an identification of the separate modifications of consciousness and of the mental faculties with mind itself. An act of remembrance is an affection of mind—the universe, according to Spinozism, is an affection of God. In one place, our philosopher says that all things have the same relation to God, as the property of having the sum of its angles equal to two right angles has to the triangle. This is something more than the old doctrine that "God is the soul of the world." Ideas are termed "modes of thought," and they must have objects. The only object to the Divine thought is the Divine existence or essence, and all that flows from it: he is eternally employed in contemplating himself alone. What God thinks as cogitative, he does as extended, and *vice versâ*. His thought and his act are but one phenomenon under a twofold aspect. The circle is a mode of God as possessing extension; the idea of a circle is the corresponding mode of God as thinking. The Divine Being himself also thinks of his own thoughts, by self-consciousness; and all man's ideas are only determinate portions of the ideas of God. As to finite body and spirit, they are only forms of extension and thought, modes, or (as Spinoza sometimes calls them) "portions" of the Divine extension and the Divine thought. Body and soul appear to be two, but they are not,—they are only two aspects of one thing; aspects of the one substance, in its extension and its thought. As though to mystify the doctrine of finite spirit still further, our author asserts, anticipatorily of some of the modern German speculations, that "the soul is the sum of the ideas which are brought together at any one moment." It is nothing more than a succession of ideas, which are only the result of different changes taking place in the body.

Spinoza assigns to affections of the body all our ideas. "We know our own bodies only by means of their affections, and external bodies by the affections of our own; and we know our own spirits only by means of the idea of those affections."* Nevertheless, all man's ideas, whether derived or intuitive, are determinate portions of the ideas of the Deity, and are produced by necessity. This statement does not well cohere with another, in which he says that the mind has the power of regulating the ideas which are its constituent elements, and of controlling their development. Here we may observe that there is a considerable discrepancy between the metaphysical and the moral part of the system. In the former, Spinoza speaks the language of necessity and fatalism; in the latter, he ascribes to man a certain sort of command or influence over his ideas: and he seems to found his whole system of practical morality on the notion of liberty or power exerted by man over his trains of thought. We must not now attempt to dwell further on the extent of this inconsistency.† A similar obscurity is usually admitted to rest on his account of the process by which general ideas are produced from those which are individual.

Notwithstanding the apparent rigour of Spinoza's deductions, the reader will by this time be prepared to see that their conclusiveness towards establishing the system is in appearance only, not real. Many chasms in reasoning which require to be filled up are leaped over by a bound. The whole is based on gratuitous assumptions and scholastic ambiguities, in regard to the meaning of the terms substance, extension, thought, eternity, intellect, will, etc. As to the definitions and descriptions of the Divine Being, they can hardly be said to designate a really personal God. Great inconsistencies arise, even in the outset, on a comparison of the definitions, axioms, and propositions. "Substance is conceived *per se*, and requires the conception of nothing else, in order to our forming an idea of it;" and yet "the modes of substance are its affections, by which it is conceived of"—that is, by which we form an idea of it. Again: if "attributes are the essence of substance," can we conceive of substance in any other way than through its attributes?—or, would Spinoza say that the attributes are the substance? If so, why make the distinction?‡ But we must refer the reader to the first half-dozen pages of the "Ethica" for further illustration.§ He will often find that Spinoza, where he seems to prove, is only ringing incessant changes on terms; and that, although his dogmas bristle with an imposing array of geometrical forms, the remark of M. Saisset, his most recent editor, is true:—"Spinoza

* "Ethica." Part II. † See the remark of Jouffroy; p. 317.

‡ Def. III., IV., V. "Ethica." Part. I.

§ See Def. I., VIII., Comp. Prop. I. and Def. IV. V.; also Prop. III. and Ax. V.; also Prop. VII. and Def. VIII.

does not demonstrate his system—he only developes it." Leibnitz was clearly of the same opinion, and he often condemned the system in strong terms.*

Widely as Spinoza departed from the philosophy of Descartes, it is evident that he was led to his own views by his profound study of the Cartesian principles. Descartes relied on man's clear and distinct idea of a Perfect Being, as an irrefragable proof that there is one; Spinoza conceived that he had an equally valid idea of his pantheistic substance. Descartes sought to deduce, *à priori*, all the phenomena of nature necessarily from his idea of the nature and attributes of the first cause; Spinoza equally deduced them from his "clear" (intuitive) idea of the one substance. Descartes rejected final causes from philosophy; and Spinoza says they are but human fictions, and laughs at those who imagine that eyes were designed for seeing, or the sun for giving light.† Descartes represented the universe as a machine that might go on for ever, mechanically, the same quantity of motion ever remaining in it; Spinoza represented it as always having (as he inaccurately expresses it) the same proportion of motion to rest in it, and as perpetually moved without a free power, and only by necessary laws.‡ Descartes unguardedly asserted that extension alone constitutes the essence of matter; whence Spinoza saw that it followed that matter must be infinite, eternal, and necessary, since space (its essence) is so. No doubt, however, Descartes would have disowned the inferences which Spinoza so boldly drew from his philosophical principles.§

* Leibnitz was misunderstood by some as leaning to Spinozism. If there were not ample evidence to the contrary running through his works, it would be sufficient to refer to some "Critical Remarks on Spinoza" from his pen, in an original manuscript recently found in the Royal Library at Hanover. The piece is very learned, though somewhat desultory; but it touches on some of the main points of Spinozism. Among other objections, Leibnitz remarks: "Spinoza says the soul may change at any moment, because with the changes of the body there is a different idea of the body, as though the soul were an idea." "He considers creatures as evanescent modifications." "Spinoza begins where Descartes left off, in *naturalism*." "There is a medium between chance and necessity, namely, free will; this Spinoza denies." "He attributes duration to the soul only during the duration of the body: he thinks the soul perishes with the body." "He says that memory and imagination disappear with the body." We hardly think Spinoza would have endorsed this latter statement in full; his idea seems that of a kind of absorption into the One-all, though this said little for the continued personality of man. See "Refutation of Spinoza," by Leibnitz. Edited by the Rev. O. F. Owen. 1855.

† "Ethica", Part I., Prop. XXXVI.

‡ Ibid., Part II., Prop. XIII., lem. 3.

§ If matter be infinite and necessary, says Spinoza (and such it must be in his system of the universe and the Deity), then it must be one and indivisible. "This cannot be denied by those who reject the possibility of a *vacuum* (as Descartes did); for if matter could be divided so that its parts should be really distinct, why might not one part be annihilated, the rest remaining connected with each other as before?

The speculations of Spinoza have proved a fertile germ of the Pantheism which has prevailed during the last seventy years, under various and changing phases, among the Germans; sometimes assuming a purely idealistic form, at others involving a naturalism which identified matter and mind as one, and amounted to a species of Spinozism. Hamann, Herder, and Novalis, all tended towards the subsequent views of Schelling. Novalis terms Spinoza "a God-intoxicated man;" and his own speculations were a Spinozism modified by Fichteism. Schelling's "One Absolute" is at one time objective *(natura naturata)*, at another subjective *(natura naturans)*: this is little else than Spinoza's universal Substance. Schelling relies on *intellectual intuition* as the infallible organ of truth. In the idea of the Absolute, this intuition sees all things—it points them out in the Absolute, and so may be said to construct them; and in so constructing them, the particular is presented under its absolute form, brought back to its eternal idea: so Schelling. Spinoza, in similar language, says: — "The eyes of the mind, by which it sees and takes cognizance of things, are the demonstrations themselves:" (*i.e.*, we suppose intuition is demonstration.) Spinoza adds: "we perceive ourselves, and experience ourselves thus to be eternal."* Among the pantheistic theories of the moderns, that of Schelling, no doubt, most nearly resembles Spinozism; being objective, and maintaining the real existence of the Deity, who is not a mere ideal creation of the *ego*, as in the original "*Ichlehre*" of Fichte, which was an altogether subjective idealistic pantheism. In its earlier form, the philosophy of Schelling† was as completely pantheistic as that of Spinoza, and did not differ from it in any fundamental principle; though in his subsequent developments, Schelling emerged from this absolute Pantheism, and spoke of God as a Being possessed of freedom, and a distinct personal existence above and beyond the world; and of man as having a separate existence from that of his Creator. Both the systems, that of Spinoza and that of Schelling, are to be distinguished from the absolute idealism of the Hegelian Pantheism, which regards thought as the only true existence, and reduces since, of things which are really distinct from each other, the one can exist, and remain in its state, without the other." "If any one part of matter were annihilated, all extension would vanish with it." Thus did Spinoza employ and strain the Cartesian principles to uphold his own doctrine. *Vide* "Ethica." Part II. Prop. XV. Schol. Epistle IV. ad Oldenburgh.

* Mentis enim oculi quibus res videt observatque, sunt ipsæ demonstrationes: sentimus et experimus nos æternos esse. "Ethica, Lib. V.," Prop. XXIII. Schol.

† Herr Schelling, in seiner früherer Periode, wo er noch ein Philosoph war, nicht im Geringsten von Spinoza unterschied. *Der Salon*, von H. Heine. Band II., s. 121. 1834.—Die Lehre des Spinoza, und die Naturphilosophie, wie sie Schelling in seiner besseren Periode aufstellte, sind wesentlich eins und dasselbe. —Ibid., s. 267.

the Deity to a mere development of thought, in the human consciousness.

The moral tendency of Spinoza's opinions has led to strong condemnation of his system, independently of the unsoundness of its basis and superstructure as a piece of argument. Dr. Samuel Clarke's celebrated "Demonstration of the Being and Attributes of God," was especially directed against it. Both Spinoza and Clarke set out from the same point—necessary existence and necessary causation; and both speak in very similar terms of the relation of time and space to the Deity.* Clarke established natural religion on the universally admitted principle, that "something must have existed from all eternity;" which, in Spinoza's system, was blended with his doctrine of one all-absorbing substance. Clarke also strenuously rebutted Spinoza's fatalistic theory of divine and human liberty—which, as we have seen, amounted to the denial of any such thing as moral freedom.

Agreeably to a widely-extended impression of the character of Spinoza's philosophy, Clarke speaks of him as "the most celebrated patron of Atheism in our time."† This would seem to imply that he was an avowed unbeliever in a God; but Dr. Clarke could not mean this, as he had closely studied Spinoza's writings. In fact, he was, speculatively, not an Atheist, but an *Acosmist;* for he denied all real existence to the universe, and assigned all real existence to the Deity. "Far from being an Atheist," says M. Cousin, "Spinoza had such a view of God that he lost the view of man. Nothing finite appeared to him worthy of the name of existence, and with him there is no real being but the Eternal."‡ It would be unfair to Spinoza not to admit that he even utters many sentiments which involve much more than a mere philosophical admiration of infinite power, omnipresence, and eternal duration. As a single example, he says, in one place: "I have expressly asserted that the sum of the divine law, which is divinely inscribed in our mind, and the main precept of that law, is, to love God as the chief good."§ The life of Spinoza, and his character in the families in which he was domesticated, do not indicate that he realized in himself what we cannot but think was, nevertheless, the *tendency* of his Pantheism. This, however, is no uncommon fact in the history

* Clarke appears to have derived the idea of regarding finite space and infinite duration as attributes of the self-existent Being, from a passage in a Scholium of Newton's "Principia." Durat (Deus) semper, et adest ubique; et existendo semper et ubique *durationem et spatium constituit.*

† "Demonstration." 1716. p. 26.

‡ "Fragmens Philosophiques." Paris. 1838.

§ "Expresse dixi legis divinæ quæ menti nostræ divinitus inscripta est, summam, ejusque summum præceptum esse, Deum ut summum bonum amare." Epistol. 49.

of the human mind; happily, men are often better than their systems, and cannot see the direction of the road they point to. But if Spinoza has been branded as an Atheist, without due qualification, and sometimes stigmatized as a sort of monster of impiety, certain it is that he has on the other hand been as much lauded, as though he had been one of the first saints in the Romish calendar.* Even some of those, however, who have been most anxious to do him justice, have not failed to admit that whatever might be the effect of his system on himself, "his doctrine amounted to Atheism, or little better."† Surely it is so; for Spinoza's God is identified with man and the universe, all being bound together by an inexorable necessity; so that his Infinite Power is no more directed by design and choice, than the motion of a steam-engine or a railroad; and, being thus destitute of all liberty or free will, and even of all intelligence that is not in some mystical way identical with unlimited power, he can possess no true and real personality.

The failure of men of such extraordinary mental stature as Descartes, Leibnitz, and Spinoza, to establish a satisfactory system of metaphysical knowledge on some of the most interesting subjects that can engage the human mind, shows one at least of two things: either a just metaphysical philosophy is impossible, or their method of aiming to attain it is wrong. Now, the method, or fundamental theory of human knowledge advocated by the three great men above-named was essentially the same. It amounted to the doctrine of an infallible intuition: clear and distinct ideas, well-ascertained to be such, must lead to truth; and yet, to what issues has this theory conducted? It led Descartes to his vortices, Leibnitz to his monads, and Spinoza to his universe-God. What caution, then, is required—what

* *Vide* Paulus's Spinoza. Jena, 1803.—M. Cousin says,—"Sa vie est le symbole de son systême. Adorant l'Éternel, sans cesse en face de l'Infini, il a dédaigné ce monde qui passe; il n'a connu ni le plaisir, ni l'action, ni la gloire, car il n'a pas soupçonné la sienne. Spinoza est un Mouni Indien, un Soufi Persan, un moine enthusiaste; et l'auteur auquel réssemble le plus ce prétendu athée, est l'auteur inconnu de *l'imitation de Jésus Christ.* "Fragm. Philos."

Göthe says, "The great mind that had so great an influence on mine was Spinoza's. After I had looked round the world in vain for means of shaping my strange moral being, I fell at last on the 'Ethics' of this man. I found there a calm to my passions; it seemed to open to me a wide and free view over all the sensuous and moral world. But what particularly riveted me was the boundless disinterestedness that beamed forth from every sentence," etc.—"*Göthe: Dichtung und Wahrheit,*" p. 14.

Schleiermacher, a Lutheran clergyman, says, "Offer up with me a lock of hair to the holy but despised Spinoza! The mighty spirit of the universe penetrated him; the Infinite was his beginning and end. He was filled with religion and religious devotion; and on this account he stands alone, elevated above a profane world, without disciples, and even without citizenship."—"*Rede über die Religion,*" page 47.

† "Biog. Hist. of Philos.;" article Xenophanes. See Vol. i. p. 85.

freedom from the *ignes fatui* of imagination, and from the egoism of association and pre-conceived notions, before we can safely apply so limited a power as the intuitional faculty of man, for seizing on truth! What fallacies may underlie assumptions which are supposed to be proof against all objections; and how easily, in a chain of argument professing to be constructed on the principles of an infallible logic, may an unsound link become interpolated, and vitiate the whole! Again, may we not safely say, that the aim of metaphysics has often been extravagantly high? The limits of the human faculties have been forgotten, and the failure has been proportionably signal; in no cases more so than in the successive pantheistic systems of modern times, from Spinoza to Hegel.

---

